# Comparison of Silicon Nanocrystals Prepared by Two Fundamentally Different Methods

**DOI:** 10.1186/s11671-016-1655-7

**Published:** 2016-10-03

**Authors:** Ondřej Cibulka, Christoph Vorkötter, Adam Purkrt, Jakub Holovský, Jan Benedikt, Kateřina Herynková

**Affiliations:** 1 Institute of Physics, Academy of Sciences of the Czech Republic, Cukrovarnická 10, 162 00, Prague, Czech Republic; 2 Institute for Experimental Physics II: Coupled Plasma-Solid State Systems, Ruhr-University Bochum, 44780 Bochum, Germany; 3 CTU Faculty of Electrical Engineering, Technická 2, 166 27 Prague, Czech Republic

**Keywords:** Silicon nanocrystals, Electrochemical etching, Low-pressure plasma, Photoluminescence, Size distribution, Surface passivation

## Abstract

This work compares structural and optical properties of silicon nanocrystals prepared by two fundamentally different methods, namely, electrochemical etching of Si wafers and low-pressure plasma synthesis, completed with a mechano-photo-chemical treatment. This treatment leads to surface passivation of the nanoparticles by methyl groups. Plasma synthesis unlike electrochemical etching allows selecting of the particle sizes. Measured sizes of the nanoparticles by dynamic light scattering show 3 and 20 nm for electrochemically etched and plasma-synthetized samples, respectively. Plasma-synthetized 20-nm particles do not exhibit photoluminescence due to absence of quantum confinement effect, and freshly appeared photoluminescence after surface passivation could indicate presence of organic molecules on the nanoparticle surface, luminescing instead of nanocrystal core. Electrochemically etched sample exhibits dramatic changes in photoluminescence during the mechano-photo-chemical treatment while no photoluminescence is observed for the plasma-synthetized one. We also used the Fourier transform infrared spectroscopy for comparison of the chemical changes happened during the treatment.

## Background

*Silicon* as a widely available and low-cost material plays a key role in the present *micro*electronics, because the commonly used and well-mastered CMOS technology is almost exclusively based on this element [1]. However, from the point of view of semiconductor physics, silicon is an indirect band gap material which emits, unfortunately, only extremely weak photoluminescence (PL) in the near infrared region. Consequently, poor light emission hinders silicon from the use in *opto*electronic applications.

*Nanocrystalline* forms of silicon, however, due to a combination of the quantum (spatial) confinement effect in silicon nanocrystals (sized 2–5 nm) and enhanced role of the surface emit strong photoluminescence in the visible region. First observation of the light emission from so-called porous silicon (a conglomerate of silicon nanocrystals), reported in 1990 by Canham [2] and followed soon by many others [3], raised hopes to develop a CMOS compatible light source—an efficient light-emitting diode or even silicon laser.

Electrochemically etched silicon nanocrystals (SiNCs) dissolved in a xylene-based mixture of organic solvents [4] exhibit, after mechano-photo-chemical procedure, dramatic changes of the photoluminescence dynamics [5]. Optically clear suspension with almost no light scattering, extremely bright photoluminescence emission in the yellow region (580 nm) and short radiative lifetime with high PL quantum efficiency (about 20 %) is obtained after this patented treatment of the solution [6]. This might open door to an efficient CMOS compatible silicon-based light source. Nuclear magnetic resonance analysis revealed that these SiNCs are surface passivated with methyl (–CH_3_) groups instead of native oxide (SiO_2_) passivation.

The SiNCs’ preparation technology used in our laboratory—the electrochemical etching of silicon wafers followed by mechano-photo-chemical treatment—produces top-optical-grade silicon nanoparticles; however, it is relatively time-consuming and with unsatisfactory production yield. Recently we have focused our research on new much less time-consuming ways how to produce SiNCs in stabilized solutions with similar excellent optical properties and with higher production rates. A low-cost and suitable alternative method with much higher yield appears to be a low-pressure gas-phase plasma synthesis.

In the 1990s, several groups reported the formation of SiNCs using low-pressure plasmas [7]. Starting in 2004, Kortshagen [8] reported a low-pressure experimental set-up for high-throughput production of SiNCs, which consisted of two RF-powered ring electrodes around a quartz tube. This plasma process is very efficient in the transformation of silane into nanoparticles (50–80 %) and has high production rates up to 50 mg.h^−1^ of SiNCs with diameters of 3 nm or larger. It is necessary to apply hydrosilylation to those nanoparticles for surface passivation with organic molecules to obtain PL maximum in the red spectral region [9].

In this paper, we follow two different goals. At first, we apply our patented mechano-photo-chemical treatment to low-pressure plasma gas-phase synthetized SiNCs to reveal whether this process will be able to passivate SiNCs of various origin by –CH_3_ groups. Secondly, we wish to find out if 20-nm-sized originally non-luminescent SiNCs will exhibit some PL after attachment of –CH_3_ or similar radicals on their surface instead of nanocrystal core. On the other hand, no PL appearance will confirm that our mechano-photo-chemical treatment and resulting change in the surface passivation is responsible for the improved optical properties of the crystalline nanoparticles.

## Methods

### Preparation of silicon nanocrystals

Two preparation techniques were utilized for generation of SiNCs—electrochemical etching of Si wafer and low-pressure plasma synthesis. Highly porous silicon prepared by electrochemical etching consist of bigger clusters of interconnected luminescent silicon nanocrystals [10, 11], representing an example of a “top-down” method. Plasma-based synthesis belonging to so-called “bottom-up” techniques allows to prepare isolated nanoparticles with rather precise particle size control, leading to narrow nanoparticle size distribution and reduced particle agglomeration [12, 13].

**Fig. 1:**
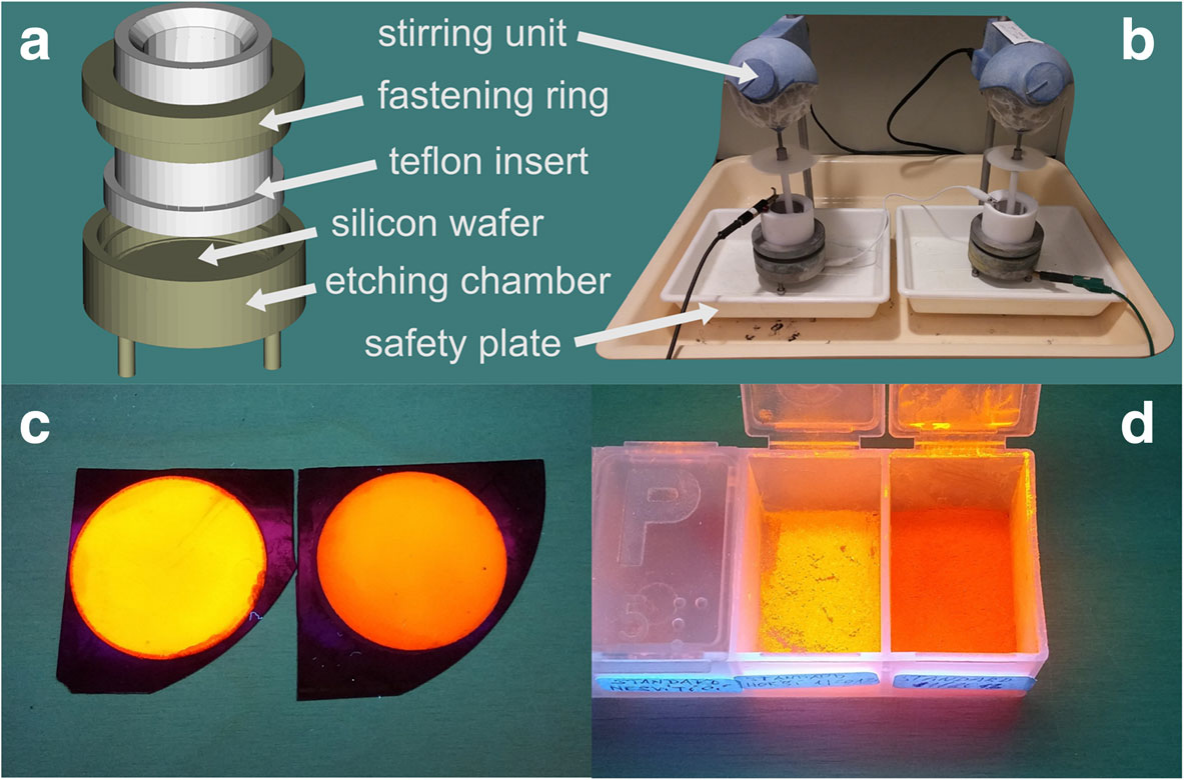
Electrochemical etching. Etching chamber (**a**) is composed of a base with a silicon wafer, teflon insert with a platinum electrode at the top and a special anti-corrosive seal at the bottom and a fastening ring. Workplace (**b**) composes from two etching chambers electrically connected in a series. Solution of each chamber is permanently stirred by a teflon stirrer. Porous silicon layer on top of the etched wafers (**c**) and scratched powder of SiNCs (**d**) exhibit bright PL in yellow/orange region

Highly porous silicon were prepared by 2-h anodic electrochemical etching of p-type <100 > crystalline silicon wafer (resistivity of 0.06–0.10 Ω·cm^−1^, doped with boron) in an aqueous solution consisting of 13 ml of 50 % HF acid and 37 ml of ethanol at current density of 16 mA/cm^2^ (Fig. 1). Ethanol is added as a surfactant to reduce the surface tension of the etching solution and to enable full wetting of the silicon substrate. Powder of porous silicon was then mechanically scraped from the substrate [4, 14].

**Fig. 2:**
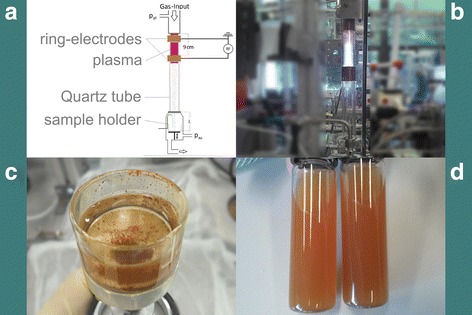
Low-pressure gas-phase plasma reactor. The plasma reactor (**a**) is composed of a quartz tube, two RF electrodes, gas inlet, and a sample holder. Plasma is generated between two RF electrodes (**b**) and SiNCs are collected in a glass sample holder (**c**). Final stage of production of the plasma-synthetized SiNCs (**d**)

Silicon nanocrystals prepared by plasma synthesis have been produced using silane (SiH_4_) as precursor using a non-thermal plasma reactor (Fig. 2) design based on the system as developed by Kortshagen and Mangolini and described in [15]. The reactor consists of a 30-cm-long quartz tube with an outer diameter equal to 2.54 cm, connected at both ends to ultra-vacuum fittings and evacuated using a Roots pump. The plasma pressure for the silicon nanoparticle generation is 400 Pa. The plasma is generated between a ring electrode wrapped around the tube and one of the vacuum flanges, typically the one on the pump side of the system. The electrical input power is supplied using a 13.56 MHz power supply. The copper ring electrode is 2.54 cm wide, and kept at a distance of 9 cm from the nearest metal flange. Silane is supplied from a compressed tank at a concentration of 1.37 %, balance argon. The particle size depends on applied power, electrode distance, and admixture of hydrogen. Different sets of conditions lead to three particle sizes – 5, 20 and 70 nm. Although the 5 nm particles exhibit weak luminescence as shown in inset of the Fig. 4a, we selected 20 nm parti-cles in size for our experiments, which are originally non-luminescent, to demonstrate if those SiNCs will exhibit some PL after the surface passivation instead of nanocrystal core. On the other hand, no PL appearance will confirm that our mechano-photo-chemical treatment and resulting change in the surface passivation is responsible for the improved optical properties of the crystalline nanoparticles.

### Post-treatment

Both kinds of SiNCs, electrochemically etched and plasma synthetized, were dispersed into a xylene-based mixture of organic solvents which contains mainly xylene isomers, ethylbenzene and isopropylbenzene (discussed in more detail elsewhere [16]). During permanent magnetic stirring, both colloidal suspensions were periodically illuminated for 5 weeks by Kimmon continuous wave He-Cd laser (325 nm, 2 mW), two times per week for 20 min. This long-term procedure is necessary for activating of the organic passivation and sufficient cover of the SiNCs by –CH_3_ groups as was discussed in our previous works [4, 16]. A control sample (mixture of the organic solvents without any SiNCs) was also treated by this procedure.

After finishing of the mechano-photo-chemical treatment, the electrochemically etched SiNCs were precisely filtrated to eliminate bigger clusters of nanocrystals which would affect the dynamic light scattering (DLS) measurements of sizes. The solution with plasma-synthetized SiNCs was milky due to much bigger amount of nanoparticles and their partial agglomeration, see Fig. 2d. The preliminary filtration of this sample by sedimentation leads to a monodisperse solution.

### Optical and structural properties of the silicon nanocrystals

Silicon nanocrystals of both types were compared in several criterions. The size of the nanoparticles, clusters or individual nanocrystals, was measured in colloidal solutions by DLS method by a Malvern Zetasizer Nano device.

Steady-state photoluminescence was measured under continuous wave excitation by a Kimmon He-Cd laser, excitation wavelength 442 nm. The PL was collected by an optical fiber and detected using a Horiba Triax 320 spectrograph coupled with an Andor spectroscopic CCD camera. An edge filter at 442 nm was used for cutting of the laser line from the detection and all measured spectra have been spectrally corrected.

The infrared absorbance spectra were measured using a Nicolet Nexus 870 FTIR spectrophotometer equipped with custom-made attenuated total reflectance (ATR) accessory (prisms of various shapes). A ZnSe prism 50 mm long, 20 mm wide, and 3 mm thick was used to enhance absorptance by achieving tens of internal reflectances. The process of solvent drying was tracked by periodical spectra collection. This allowed identification of oxidation processes.

## Results and Discussion

**Fig. 3:**
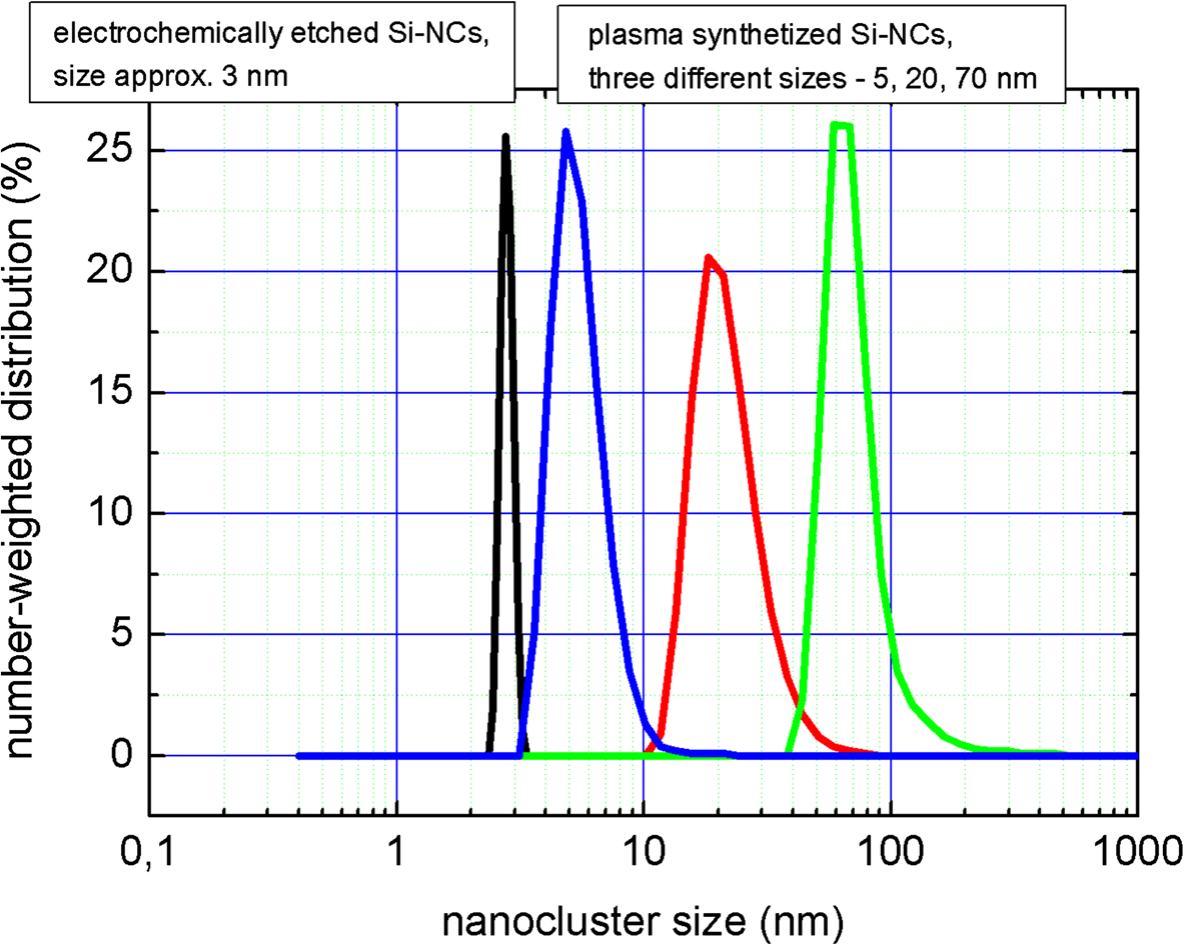
Size measurements. DLS measurements of electrochemically etched sample show SiNCs 3 nm in diameter while similar investigation of plasma-synthetized particles shows several different sizes—5 nm (*blue curve*), 20 nm (*red curve*), and 70 nm (*green curve*). SiNCs of 20 nm in size were used in this study

The size analysis of nanoparticles prepared by both methods is shown in Fig. 3. The nanocrystals’ mean size of the electrochemically prepared SiNCs is (after precise filtration) about 3 nm. The size of silicon nanocrystals prepared by plasma-based synthesis depends on distance between electrodes, dilution of silane, and value of applied power. It can vary from several nm up to tens of nm, as shown in Fig. 3. The size of SiNCs under study was about 20 nm which should indicate that those SiNCs will show no PL because quantum confinement effect is not yet pronounced in such big nanocrystals. This is an advantage if one wants to distinguish PL from passivated surface of nanocrystals and from their core.

**Fig. 4:**
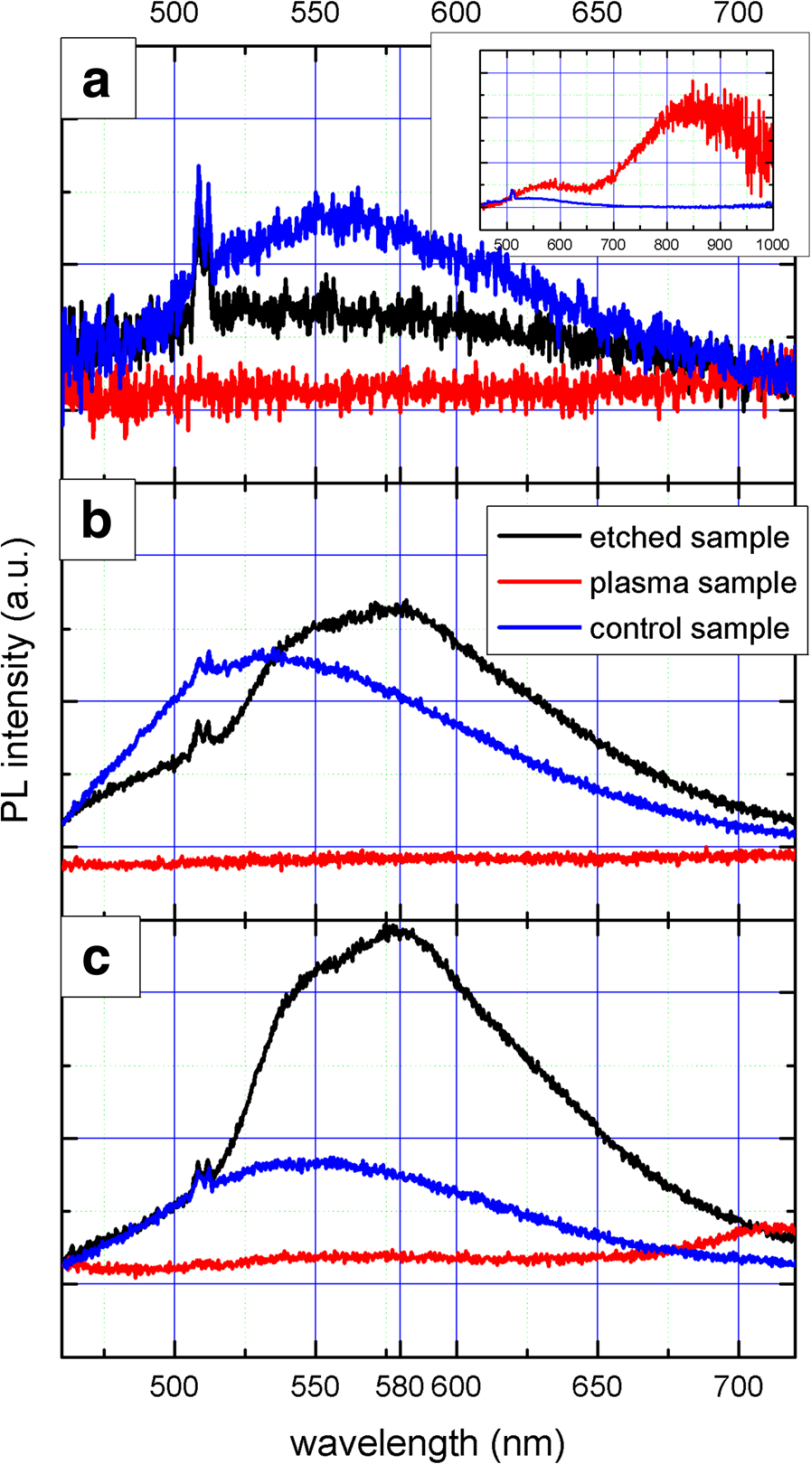
Time evolution of photoluminescence. Panel **a** shows initial state of PL. Electrochemically etched (*black curve*) and control samples (*blue curve*) exhibit weak PL at 550 nm while plasma-synthetized sample with 20-nm particles (*red curve*) exhibit no PL. *Inset* of panel **a** shows PL of 5 nm plasma-synthetized particles which are not study in this paper. Panel **b** shows PL after 2 weeks of treatment. PL of the control sample slightly increases; new peak at 580 nm appears in PL of the electrochemically etched SiNCs while plasma-synthetized ones exhibit no PL all the time. Panel **c** shows PL after 5 weeks of treatment. PL of the control sample remains constant; the peak at 580 nm strongly increases in the case of electrochemically etched SiNCs but plasma-synthetized ones exhibit constantly no PL

The time evolution of the PL measurements during the mechano-photo-chemical treatment is shown in Fig. 4. Initial state of PL in Fig. 4a shows that the control sample (blue curve) exhibits PL at a 550 nm while the selected 20-nm plasma-synthetized SiNCs (red curve) show no PL in the same region. Inset in Fig. 4a shows the PL of 5-nm plasma-synthetized particles which are not study in this paper. PL of the control sample slightly increases during 5 weeks of periodical illumination by 325-nm wavelength of He-Cd laser (Fig. 4b, c) while the 20-nm plasma-synthetized SiNCs exhibit no PL change even after the whole procedure has been finished.

On the other hand, the photoluminescence of the electrochemically etched SiNCs (black curve) exhibits very low intensity at the starting point with a peak at the same wavelength as the control sample (Fig. 4a). However, a new peak at 580 nm appears during periodical treatment and increases dramatically in intensity in the course of after several weeks (Fig. 4b, c).

Electrochemically etched single SiNCs sized around 3 nm thus have reproduced behavior of changed surface passivation described in our previous paper [16]. Exchange of the surface layer of native SiO_2_ for methyl groups from the chemicals in the solvent leads to the appearance of the new peak at 580 nm and increase of its PL intensity [17].

An interesting effect is that, even if the size of the plasma-synthetized SiNCs of about 20 nm is too big to show any luminescence. However, their presence in the solvent caused also quenching of the solvent PL itself. This effect could have several reasons. It has been reported that the excited singlet state of the organic dyes is efficiently quenched by the amino acid tryptophan via photoinduced electron transfer [18]. A fluorescence quenching was also observed for the organic dye molecules chemically attached to differently sized gold nanoparticles from the sizes of 1-nm radius [19]. The specific reason in our case can be tentatively attributed to chemical changes on the nanocrystals’ surface and should be investigated in more details.

**Fig. 5:**
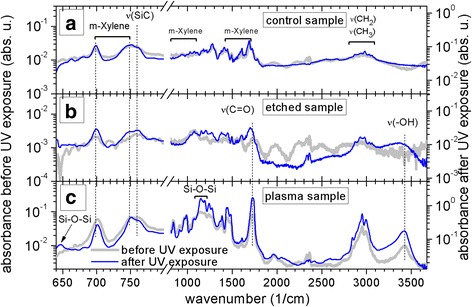
FTIR measurements. Infrared spectra of nanocrystals after complete drying of the solvent, taken in the attenuated total reflectance mode. **a** Control sample. **b** Etched sample. **c** Plasma sample

The FTIR-ATR spectrum of dried solvent is in Fig. 5a. The two peaks at around 700 and 750 cm^−1^ likely correspond to meta-xylene, i.e., the organic solvent itself [20]. The electrochemically etched SiNCs shown in Fig. 5b have infrared spectra similar to the solvent spectrum. This is mainly due to the fact that the amount of nanocrystals in the sample was relatively low. On the other hand, the infrared spectrum of plasma-synthetized sample contains features around 650 cm^−1^ and in the range 1100–1200 cm^−1^ that are neither seen in the above two spectra, neither before drying the solvent (not shown). We assign this structure to Si-O-Si vibrations [21]. The plasma-synthetized sample contains much higher concentration of SiNCs and they are rather strongly oxidized. Also hydrocarbons attached to the nanocrystals are much more oxidized as seen from the stretching modes C=O at around 1700 cm^−1^ and stretching of –OH group at around 3400 cm^−1^. The peak of interest—the Si-C stretching mode at 762 cm^−1^ [22, 23]—is partly overlapping with the meta-xylene peak at 750 cm^−1^ and it is therefore difficult to clearly observe its changes. Nevertheless, comparing the ratio of the peak at 762 cm^−1^ to the one at 1700 cm^−1^ for both samples, we can argue that the electrochemically etched sample has more Si-C bonds and is less oxidized than the plasma-synthetized one.

## Conclusions

Two fundamentally different methods were used for fabrication of the SiNCs—electrochemical etching and low-pressure gas-phase plasma synthesis. Both types of SiNCs were treated by a mechano-photo-chemical method to passivate their surface by methyl groups. The advantage of plasma synthesis, tuning of the SiNC size, was used for fabrication of the nanocrystals 20 nm in diameter which, however, exhibit no PL due to the absence of quantum confinement effect. If some PL appeared after organic passivation of those SiNCs, it could indicate that this PL originated in organic molecules on the surface of nanocrystal and not in nanocrystal’s core.

Optical and structural properties of both types of nanoparticles were studied. Electrochemically etched sample shows dramatic increase of PL at the wavelength of 580 nm during 5 weeks of treatment. Passivation of the SiNCs surface by methyl groups, evidenced by the FTIR spectra, causes this change in PL behavior. On the other hand, big nanoparticles prepared by plasma-based synthesis show immediate PL quenching of solvent and no change during the whole treatment process. Though the FTIR spectra of this sample indicate partial surface passivation by methyl groups the absence of any PL indicates that organic molecules possibly grafted on the nanocrystal surface are not able to generate any PL which is in agreement with our recent findings [24].

Our results open several new tasks. One of them is different PL behavior of the solvent after dissolving SiNCs of different origin. Adding of the plasma-synthetized SiNCs to the solvent totally quenches its PL. Actual reason should be investigated in more details. Secondly, experiments with crystalline plasma-synthetized SiNCs with size of 5 nm and, therefore, with strong quantum confinement are being prepared.

## Abbreviations

DLS: Dynamic light scattering
FTIR: Fourier transform infrared (spectra)
HF: Hydrofluoric acid
PL: Photoluminescence
SiNCs: Silicon nanocrystals
